# Antifungal and Antioxidant Activities of the Essential Oil from *Angelica koreana* Nakai

**DOI:** 10.1155/2014/398503

**Published:** 2014-08-12

**Authors:** Junghyun Roh, Seungwon Shin

**Affiliations:** College of Pharmacy, Duksung Women's University, Seoul 132-714, Republic of Korea

## Abstract

*Purpose*. The purpose of this study is to determine the antifungal and antioxidant activities of the essential oil from *Angelica koreana*. *Methods*. Essential oil was obtained from the dried roots of *A. koreana* by steam distillation, and its composition was identified by gas chromatography and mass spectrometry (GC-MS). The minimal inhibitory concentrations (MICs) of the oil fraction and its main components were determined by broth dilution assay using common pathogenic *Aspergillus* and *Trichophyton* species. The combined effects of the oils with itraconazole were evaluated using a checkerboard titer test. In addition, 1,1-diphenyl-2-picryl-hydrazil (DPPH) free radical scavenging, nitrite inhibition, and reducing power were determined to assess the antioxidant activity of this oil. *Results*. The essential oil fraction and its main components showed inhibitory activity against all of the tested fungi, with minimal inhibitory concentrations (MICs) of 250–1000 *μ*g/mL. Furthermore, this oil exhibited synergism when combined with itraconazole. *Conclusion*. In the treatment of infections caused by *Aspergillus* and *Trichophyton* species, combining itraconazole with either *A. koreana* essential oil or its main components may reduce the minimum effective dose of itraconazole required and, thus, minimize its side effects. In addition, this oil is a promising source of natural antioxidant agents.

## 1. Introduction

The roots of* Angelica koreana *Nakai (Apiaceae) are used in oriental medicine for treatment of cold, especially for patients that present with symptoms such as chills, fever, headaches, body aches, and pains [1]. The acaricidal toxicity and hypopigmenting activity of the components of* A. koreana* have been reported [2, 3]. Essential oils are one of the most promising sources for the development of new natural antimicrobial and antioxidant agents, even though these oils appear to have relatively mild activities compared to commercial, synthetic agents [4–7].

*Aspergillus *species cause a number of severe diseases, both in normal and immunocompromised hosts, encompassed under the name aspergillosis and including allergic disease, saprophytic disease, superficial infections, and invasive infections [8–10]. Among the* Aspergillus *species,* A. fumigatus *is the most common human infectious agent, followed by* A. flavus, A. niger, *and* A. terreus *[11–13].* A. versicolor* produces many toxic compounds, which can cause severe symptoms in humans and animals infected through inhalation or other forms of contact with debris or spores [14, 15].

*Trichophyton *species are pathogenic fungi causing superficial mycoses, commonly known as tinea infections, in various tissues of humans and other animals [16].* T. rubrum *is a predominate cause of dermatophytosis, followed by* T. mentagrophytes *and* T. tonsurans *[17–20].

Antioxidants are used as food additives to protect humans and food from undesirable oxidative reactions. The antioxidant activities of plant compounds have been increasingly investigated in recent decades for the development of new natural antioxidative agents [21, 22]. Plant essential oils are a promising source of natural antioxidants [23, 24].

Here, to specifically determine whether stable and safe antifungal agents could be developed from the essential oil of* Angelica koreana*, its inhibiting activity against common pathogenic fungi (three* Aspergillus* fungi and five* Trichophyton* species) was investigated using a microbroth dilution test. In addition, we determined the synergistic effects of* A. koreana* root essential oil fraction and its main components with itraconazole using checkerboard microtiter tests.

To estimate the value of this oil as an antioxidant agent, 1,1-diphenyl-2-picryl-hydrazil (DPPH) free radical scavenging, nitrite inhibition, and reducing power, the corresponding experiments were performed.

## 2. Materials and Methods

### 2.1. Sample Preparation

The essential oil was obtained by steam distillation of the dried roots of* Angelica koreana*, which was cultivated in Jinbu, Gangwon-do, Korea.

### 2.2. Fungal Strains

*A. flavus* KCCM11453,* A. flavus* KCCM11899,* A. flavus* KCCM11910,* A. fumigatus* KCCM60027,* A. fumigatus* KCCM60331,* A. terreus* KCCM12067,* A. terreus* KCCM12709,* A. niger* KCCM11239,* A. niger* KCCM11240,* A. niger* KCCM11241,* A. versicolor* KCCM12714,* A. versicolor* KCCM35225,* T. tonsurans* KCCM11866,* T. tonsurans* KCCM60442,* T. mentagrophytes* KCCM11950,* T. mentagrophytes* KCCM60444,* T. rubrum* KCCM60443, and* T. rubrum* KCCM60450 were obtained from the Korean Culture Center of Microorganisms (KCCM). They were cultured in yeast and malt extract broth for 48 h at 28°C. The turbidity of the* Aspergillus* cell suspensions was measured at 625 nm and adjusted with medium to match the 0.5 McFarland standard. The inocula suspensions of* Trichophyton* species were prepared using the method as described by the Clinical and Laboratory Standards Institute (CLSI) [25]. The microconidia were counted on a hemocytometer and diluted to a final concentration of ca. 0.5 × 10^4^ CFU/mL.

### 2.3. Gas Chromatography-Mass Spectrometry (GC-MS) Analysis

GC-MS analysis was carried out using a Hewlett Packard (HP) 5973–6890 system equipped with an HP-5MS capillary column. The oven was programmed with an initial temperature of 50°C and heated at a rate of 2°C/min to 170°C.

### 2.4. Isolation of m-Cresol and Sabinene

The essential oil fraction (10 g) of* A. koreana* was subjected to column chromatography over a silica gel and was eluted with the solvent, toluene-ethyl acetate (95 : 5). The fractions were combined based on thin layer chromatography (TLC) pattern in order to yield subfractions designated A1–A10. Subfraction A5 was further purified by column chromatography over a silica gel and was eluted with toluene-ethyl acetate to give 21 mg of m-cresol (99.8%). Sabinene (91.5%) was obtained by repeated fractional distillation with the hydrocarbon fraction of this oil. The ultraviolet spectrometry (UV), mass spectrometry (MS), ^1^H-nuclear magnetic resonance spectrometry (NMR), and ^13^C-NMR results were identical to those of the authentic standard sample (Sigma, USA).

### 2.5. Determination of the Minimal Inhibitory Concentrations (MICs)

The MICs of the antifungal agents against the various fungi were determined using a modified broth microdilution test as described by the CLSI. The essential oils of* Angelica koreana*, m-cresol and sabinene, were serially diluted with ethanol to obtain 0.0125~16 mg/mL solutions, and 10 *μ*L/mL of Tween 80 was added to each solution. After shaking, 100-*μ*L aliquots of these solutions were added to the wells of a 96-well microtiter plate. Itraconazole was similarly diluted in dimethyl sulfoxide (DMSO) to generate a series of concentrations ranging from 128 to 0.25 *μ*g/mL per testing well. A 100-*μ*L suspension of each organism was adjusted to a final concentration of ca. 0.5 × 10^4^ CFU/mL and then added to the individual wells and cultivated at 28°C. The MIC was defined as the lowest concentration that completely inhibited visible fungal growth after 48 h of incubation. Each organism was also cultured with a blank solution containing Tween 80, ethanol, and DMSO at concentrations equivalent to those in the test solutions in order to verify that these vehicles did not affect fungal growth. The tests were performed in triplicate.

### 2.6. Checkerboard Microtiter Test

Ten serial, two-fold dilutions of* A. koreana* essential oil, m-cresol, sabinene, and itraconazole were, respectively, prepared with the same solvents used in the MIC tests. Then, 5-*μ*L aliquots of each dilution were added to the wells of a 96-well plate in the vertical orientation, and 5-*μ*L aliquots of each itraconazole dilution were added in the horizontal orientation so that the plate contained various concentration combinations of the two compounds. A 100-*μ*L suspension of each fungal strain (0.4 × 10^4^ CFU/mL) was added to the individual wells, and the plate was incubated at 28°C for 72 h. Fractional inhibitory concentrations (FICs) were calculated as the MIC of the combination of essential oil/m-cresol/sabinene and itraconazole divided by the MIC of essential oil/m-cresol/sabinene or itraconazole alone. The FIC index (FICI) was calculated by summing both FICs and was interpreted as a synergistic effect when it was <0.5, as additive when it was 0.5 to 1.0, as indifferent when it was > 1.0 to 2.0, and as antagonistic when it was >2.0 [26].

### 2.7. DPPH Scavenging Effects of* A. koreana* Essential Oil

A fresh solution of 0.1 mM DPPH and two-fold dilutions of an* A. koreana* essential oil fraction (or its main component) (3.2-0.05 mg/mL, final concentration) were prepared in ethanol, and 900 *μ*L of DPPH was mixed with 100 *μ*L of the oil sample. After vortexing for 10 sec, samples were added to five wells in 96-well plates and kept at room temperature for 30 min. A decrease in absorbance (ABS) was monitored at 540 nm. DPPH radical scavenging capacity was calculated using the following equation [27]:

(1) DPPH  scavenging  effects  (%) =100×[1−{ABS  of  sample(Abs  of  DPPH−ABS  of  sample)}]

### 2.8. Nitric Oxide Scavenging Activity

Nitric oxide scavenging activity was measured using the method of Kato et al. [28]. In brief, 10 *μ*L of sodium nitrite (2 mMol) and 10 *μ*L of each serial dilution of the oil sample were mixed with 80 *μ*L of citrate buffer (0.2 Mol) and adjusted to pH = 3.0. The mixtures were incubated at 37°C for 1 h. Next, 200 *μ*L of 2% acetic acid solution was added to 40 *μ*L of each mixture in a 96-well plate. Then, 16 *μ*L of Griess reagent (1% sulfanilamide, 2% phosphoric acid, and 0.1% naphthyl ethylenediamine dihydrochloride) was added to each well. After 15 min, the absorbance of the chromophore formed during the diazotization of the nitrite with sulfanilamide and the subsequent coupling with naphthyl ethylenediamine dihydrochloride was measured at 546 nm [29].

### 2.9. Determination of Reducing Power

Reducing power was determined according to the method of Elmastas et al. [30]. Various concentrations of* A. koreana* essential oil fraction were prepared as two-fold dilutions (12.5–200 *μ*g/mL, final concentration) with methyl alcohol. Each sample (0.2 mL) was mixed with 0.6 mL of 0.2 M phosphate buffer (pH 6.6) and 0.6 mL of 1% potassium ferricyanide. The mixture was incubated at 50°C for 20 min, and 0.6 mL of 10% trichloroacetic acid was added before 10 min of centrifugation at 1000 ×g (Hanil Science, Korea). The upper layer (1 mL) was mixed with FeCl_3_ (0.1 mL, 0.1%), and its absorbance was measured at 700 nm. Higher absorbance indicated greater reductive capability.

### 2.10. Single Dose Oral Toxicity Study

This study was carried out to evaluate the single dose oral toxicity of the volatile oil fraction of* A. koreana *in male specific-pathogen-free (SPF; Central Lab Animal Inc, Seoul, Korea) ICR mice. The test substance was administered to male and female mice at doses of 500, 1,000, and 2000 mg/kg. A group of five mice of each gender (5 animals per group) was treated with each dose. Mortalities, clinical signs, and body weight changes were monitored for 7 days compared to the vehicle control group. At the end of the 7-day observation period, all animals were sacrificed according to the animal experiment guidelines of the Korean Food and Drug Administration, and necropsy findings were noted.

## 3. Results and Discussion

The essential oil fraction was obtained by repeated steam distillation of the dried roots of* A. koreana*. The content of essential oil in* A. koreana *roots was 0.28%. The composition of the essential oil fraction was analyzed by GC-MS. As listed in Table 1, 48 compounds were identified in this oil, which account for 98.72% of the total composition. Sabinene (31.85%) was the predominant component of this oil, followed by m-cresol (4.46%) and *α*-pinene (4.00%), *α*-bisabolol (3.63%) and *α*-bornyl acetate.

The MICs of the essential oil fraction, its main components, sabinene and m-cresol, and the antibiotics amphotericin B and itraconazole are presented in Table 2. In the three samples of the oil, m-cresol showed the strongest growth inhibition against most of the tested fungi, with minimum inhibitory concentrations (MICs) between 125 and 1000 *μ*g/mL. Sabinene exhibited the lowest activity, with MICs ranging from 500 to >4000 *μ*g/mL. In most of the experiments, the* Trichophyton *species showed relatively higher sensitivity to the tested* Angelica koreana* oil fraction and its two main components compared to the* Aspergillus* species. The oil fraction showed the highest potency against* T. mentagrophytes*, with MICs between 250 and 500 *μ*g/mL. However, the MICs of the oil were much higher than those of the antibiotics amphotericin B and itraconazole (MICs = 0.25–8.00 *μ*g/mL), which were used as controls. In most cases, the antibiotic activities of plant essential oils are weaker than those of synthetic antibiotics used commonly in therapy. For this reason, their application in the therapy of fungal infections is very limited. Nevertheless, in consideration of the side effects of the antibiotics, essential oils have been considered as a source for the development of new natural antibiotics. Amphotericin B is highly toxic in its conventional form and expensive in its lipidic form [31]. Itraconazole is a relatively well-tolerated drug, but it has relatively low bioavailability after oral administration and produces a range of adverse effects similar to those of other azole antifungals [26]. Checkerboard titer tests were performed in this study in order to determine the synergism created by the combination of these two groups of compounds with regard to overcoming the weak activity of the oil and to minimize the side effects and toxicity of antibiotics by lowering the dosage.

The FICs and FICIs results from these checkerboard tests are listed in Tables 3 and 4. There was significant synergism and additive effects between itraconazole and* A. koreana* oil fraction/m-cresol, with FICI values ranging from 0.26 to 1.00 against the* Aspergillus* and* Trichophyton* species. Among the tested fungi in this study,* A. fumigatus* and* A. terreus* showed the most distinctive synergism with the combination of* A. koreana* oil fraction with itraconazole, resulting in an FICI of 0.37 for both* Aspergillus* species. The FICIs between 0.65 and 1.00 in the tests with* Trichophyton* species indicated additive effects between itraconazole and* A. koreana* oil fraction/m-cresol. In particular, this combination against* T. rubrum* resulted in an eight-fold decrease in the MIC with itraconazole (FIC = 0.12) compared to that before combination with the essential oil fraction. Thus,* A. koreana* essential oil might be useful in antifungal therapy when combined with itraconazole, especially against* Aspergillus *and* Trichophyton *species.

To evaluate* A. koreana* oil as an antioxidant, DPPH free radical scavenging activity, nitrite formation inhibition, and reducing power procedures were performed with the oil fraction and its two main components using butylated hydroxyl anisole (BHA, Sigma-Aldrich, USA).

**Figure 1:**
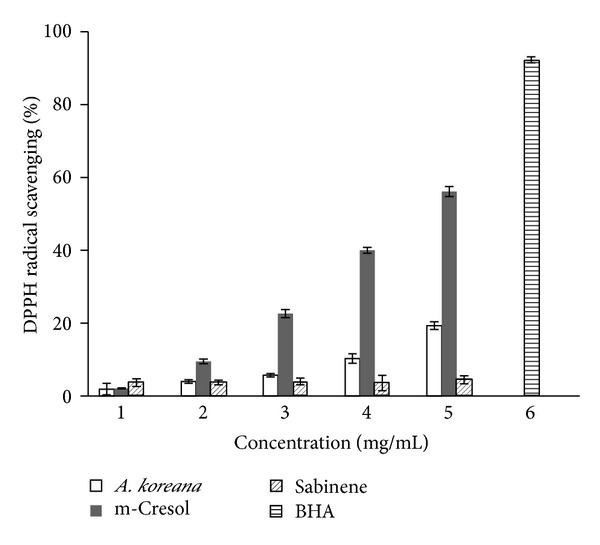
DPPH free radical scavenging activity of the essential oil fraction from the roots of* A. koreana,* m-cresol, sabinene, and BHA (control). Values are represented as the mean ± SD of triplicate measurements.

As demonstrated in Figure 1, the essential oil of* A. koreana* and its main components showed significant dose-dependent DPPH scavenging activities. One of the main components of the Angelicaessential oil fraction, m-cresol (56.12 ± 1.36%), showed greater inhibition of DPPH than did the oil fraction or sabinene (19.31 ± 1.02% and 4.45 ± 1.06%, resp.) at concentrations of 16 mg/mL. However, the results showed weaker scavenging activity than the control. At 1 mg/mL, the activity of BHA was more than 20 times higher than those of all three of the tested oil samples.

**Figure 2:**
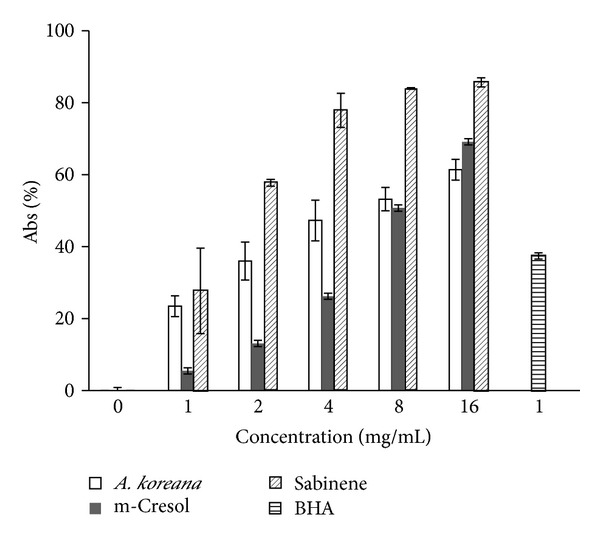
Nitrite scavenging ability of the essential oil fraction from the roots of* A. koreana,* m-cresol, sabinene, and BHA (control). Values are represented as the mean ± SD of triplicate measurements.

In nitric oxide scavenging tests, the Angelica oil fraction and its main components, m-cresol and sabinene, showed significant scavenging activities dose-dependently (Figure 2). Sabinene, exhibited the higher activity than the oil fraction or m-cresol.

**Figure 3:**
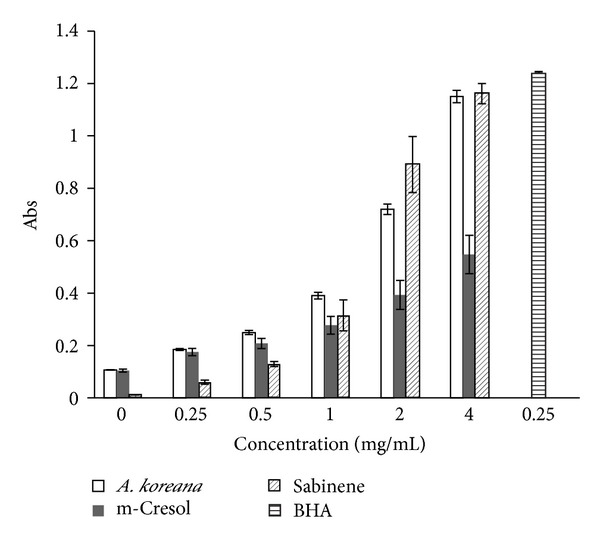
Reducing power of the essential oil fraction from the roots of* A. koreana,* m-cresol, sabinene, and BHA (control). Values are represented as the mean ± SD of triplicate measurements.

The results of reducing power assays are demonstrated in Figure 3. The* A. koreana* essential oil exhibited significant activity at concentrations of 0.25–4.00 mg/mL. Sabinene showed the strongest reducing power at 2.00 and 4.00 mg/mL. At 4.00 mg/mL, both the oil and sabinene resulted in high absorbance (0.90 ± 0.09 and 1.16 ± 1.06, resp.), indicating strong reducing power, while m-cresol showed relatively weaker activity. The absorbance tested with BHA was 1.23 ± 0.00 at the concentration of 0.25 mg/mL. This indicates that the concentration of Angelica oil needs to be five times greater than that of BHA in order to produce the same reducing power of BHA. As these results indicated, the antioxidant activity of the* A. koreana *essential oil appeared weaker than that of BHA, although its use would avoid the toxicity issues of BHA [32].

In the single dose oral toxicity study with male SPF-ICR mice, there was no death of any animal in the group administered 500 mg/kg of essential oil fraction during the experimental period. No clinical signs or body weight changes were found in relation to the tested samples. The LD_50_s of the sample were 1437.5 mg/kg for male mice. On the basis of the LD_50_ and MICs, toxicity could be excluded in the use of* A. koreana* essential oil as an anti-*Aspergillus *and anti-*Trichophyton *agent, especially in combinatorial therapy with antibiotics.

## 4. Conclusion

For the treatment of infections caused by* Aspergillus* and* Trichophyton* species, combining itraconazole with either* A. koreana* essential oil or its main components, sabinene and m-cresol, may reduce the minimum effective dose of itraconazole required and thus minimize the side effects. In addition,* A. koreana* essential oil is a promising source of new natural antioxidants. However, further studies are required to assess the potential for clinical application.

## Figures and Tables

**Table 1:** Constituents of essential oil from *A. koreana* according to GC-MS.

Compounds	RI^a^	Area (%)	Identified by
		*A. koreana *	
*α*-Pinene	905	4.00	MS^b^, GC**c**
Camphene	924	0.16	MS
*β*-Pinene	961	0.31	MS, GC
*α*-Phellandrene	1004	0.75	MS
*δ*-3-Carene	1005	2.50	MS
**Sabinene**	**1025**	**31.85**	**MS**
*β*-Thujene	1031	0.11	MS
*α*-Terpinolene	1085	0.92	MS, GC
**m-Cresol**	**1098**	**4.46**	**MS, GC**
Isovaleric acid	1107	1.61	MS
p-Cresol	1163	0.14	MS, GC
*β*-Pinone	1186	1.66	MS
*α*-Bornyl acetate	1282	2.30	MS, GC
4-Hydroxy-3-methoxy acetophenone	1290	0.54	MS
Thymol	1290	0.21	MS, GC
1-Pentalenone	1299	1.84	MS
Isoledene	1353	0.34	MS
Valencene	1370	0.20	MS
*δ*-Cadinene	1379	0.42	MS
Cedrene	1398	0.27	MS
*α*-Farnesene	1428	0.48	MS
*β*-Cubebene	1455	0.85	MS
*β*-Cedrene	1466	1.01	MS
Thujopsene	1496	0.47	MS
*α*-Muurolene	1503	0.12	MS
Sativene	1525	0.36	MS
*β*-Sesquiphellandrene	1530	0.91	MS
Elemol	1531	0.52	MS
Guaiacyl ethane	1554	1.94	MS
Viridiflorol	1580	0.62	MS
Anise alcohol	1581	0.20	MS
*α*-Amorphene	1594	0.34	MS
1,4-Methanoazulene	1618	0.85	MS
Aromadendrene	1649	0.54	MS
*β*-Eudesmol	1654	1.15	MS
*α*-Eudesmol	1659	1.14	MS
Isocomene	1673	0.41	MS
Spirodec-6-en-8-one	1688	0.61	MS
*α*-Bisabolol	1699	3.63	MS
Z-9-Pentadecenol	1734	1.34	MS
4-Butenylcyclohexene	1743	0.26	MS
Cyclohexene	1767	0.21	MS
Aromadendrene oxide	1777	0.22	MS
Cedrenol	1782	0.26	MS
Widdrol	1902	0.15	MS
3-hydroxy-4-dimethyl cyclohexaneethanol	1907	0.15	MS
*δ*-3-Carene	1963	0.29	MS
Osthole	2159	2.27	MS, GC

Total composition		98.72	

RI^a^: retention indices calculated against C_9_ to C_24_ *n*-alkanes on an HP-5MS column.

MS^b^: mass spectrum, GC^c^: Co-GC with a corresponding standard compound.

**Table 2:** MICs (*μ*g/mL) of the essential oil of *A. koreana *against *Aspergillus* and *Trichophyton* species.

Fungal strains	EOAK^a^	Sabinene	m-Cresol	Amp B^b^	Itraconazole
*A. flavus KCCM11453 *	1000	2000	500	2	1
*A. flavus KCCM11899 *	2000	4000	1000	2	1
*A. flavus KCCM11910 *	2000	4000	1000	4	2
*A. fumigatus KCCM60027 *	2000	1000	1000	8	2
*A. fumigatus KCCM60331 *	2000	1000	1000	8	2
*A. niger KCCM11239 *	2000	>4000	1000	8	2
*A. niger KCCM11240 *	2000	>4000	1000	4	2
*A. niger KCCM11241 *	1000	>4000	1000	8	2
*A. terreus KCCM12067 *	1000	4000	500	32	0.25
*A. terreus KCCM12709 *	1000	4000	500	16	0.25
*A. versicolor KCCM12714 *	250	4000	250	4	1
*A. versicolor KCCM 35225 *	250	4000	250	4	1
*T. mentagrophytes KCCM11950 *	250	500	250	NT^c^	4
*T. mentagrophytes KCCM60446 *	250	500	125	NT	4
*T. rubrum KCCM60443 *	500	500	250	NT	8
*T. rubrum KCCM60450 *	500	500	250	NT	4
*T. tonsurans KCCM11866 *	500	1000	250	NT	8
*T. tonsurans KCCM60442 *	250	500	125	NT	8

EOAK^a^: essential oil fraction from the roots of *A. koreana, *Amp B^b^: amphotericin B, NT^c^: not tested.

**Table 3:** Fractional inhibitory concentrations (FICs) and FIC indices (FICIs) of *A. koreana* essential oil in combination with itraconazole against *Aspergillus *spp.

Sample	*A. flavus * KCCM11899		*A. fumigaus * KCCM 60027		*A. niger * KCCM11241		*A. terreus * KCCM12067		*A. versicolor * KCCM 35225	
	FIC	FICI	FIC	FICI	FIC	FICI	FIC	FICI	FIC	FICI
Essential oil fraction	0.50	1.00	0.25	0.37	0.50	0.56	0.25	0.37	0.25	0.50
Itraconazole	0.50		0.12		0.06		0.12		0.25	

m-Cresol	0.25	0.50	0.25	0.50	0.25	0.26	0.50	0.62	0.06	0.56
Itraconazole	0.25		0.25		0.06		0.12		0.50	

FIC (fractional inhibitory concentration) = MIC tested in combination/MIC tested with single sample alone.

FICI = FIC of *A. koreana* essential oil or *m*-cresol oil component + FIC of itraconazole.

**Table 4:** Fractional inhibitory concentrations (FICs) and FIC indices (FICIs) of *A. koreana* essential oils in combination with itraconazole against *Trichophyton* spp.

Sample	*T. mentagrophytes* KCCM11866		*T. rubrum* KCCM11950		*T*. *tonsurans* KCCM 60443	
	FIC	FICI	FIC	FICI	FIC	FICI
Essential oil fraction	0.50	1.00	0.50	0.65	0.50	1.00
Itraconazole	0.50		0.12		0.50	

m-Cresol	0.50	1.00	0.25	0.75	0.50	1.00
Itraconazole	0.50		0.50		0.50	

FIC (fractional inhibitory concentration) = MIC tested in combination/MIC tested with single sample alone.

FICI = FIC of *A. koreana* essential oil or m-cresol + FIC of itraconazole.
